# Primary Splenic Follicular Lymphoma Presenting As Isolated Splenomegaly: A Case Report

**DOI:** 10.7759/cureus.93633

**Published:** 2025-10-01

**Authors:** Pranavi Indukuri, Steve Thomas, Pulkit Mehrotra, Sri Gayathri Shanmugam

**Affiliations:** 1 Internal Medicine, Sri Ramachandra Institute of Higher Education and Research, Chennai, IND; 2 Hematology, Sri Ramachandra Institute of Higher Education and Research, Chennai, IND; 3 Medicine, Sri Ramachandra Institute of Higher Education and Research, Chennai, IND; 4 Pathology, Sri Ramachandra Institute of Higher Education and Research, Chennai, IND

**Keywords:** follicular lymphoma, low-grade b cell non-hodgkin lymphoma, rituximab therapy, spleenectomy, spleenomegaly, splenic lymphoma

## Abstract

Follicular lymphoma (FL) is an indolent B-cell lymphoma that usually presents with lymph node enlargement; primary splenic involvement without nodal disease is exceedingly rare. We describe a woman in her late 60s with three months of early satiety, abdominal distension, and weight loss. Examination revealed massive splenomegaly without lymphadenopathy. Laboratory tests were largely normal aside from mild thrombocytopenia. Imaging positron emission tomography/computed tomography (PET/CT) demonstrated an fluorodeoxyglucose (FDG)-avid enlarged spleen with no other lesions. Bone marrow biopsy showed a benign-appearing lymphoid aggregate, and a definitive diagnosis could not be reached. The patient subsequently underwent splenectomy, and histopathology with immunohistochemistry confirmed WHO grade 2 FL confined to the spleen (Ann Arbor stage I). She was started on rituximab therapy and remains in remission on maintenance treatment.

This case highlights the diagnostic challenge of isolated splenomegaly and underscores the importance of splenectomy for both diagnosis and cytoreduction when noninvasive tests are inconclusive. Rituximab immunotherapy can achieve disease control in low-tumor-burden FL, though vigilant long-term follow-up is required.

## Introduction

Primary splenic lymphoma (PSL) is a very rare malignancy, accounting for approximately 1% of all non-Hodgkin lymphomas (NHLs) when strictly defined as disease limited to the spleen and hilar lymph nodes [1]. Most lymphomas involving the spleen are part of widespread nodal disease; thus, primary splenic follicular lymphoma (PSFL) represents an exceedingly infrequent presentation. FL is the second most common subtype of NHL and typically presents with painless lymphadenopathy. Constitutional “B” symptoms (fever, night sweats, weight loss) are uncommon in early-stage FL, occurring in only about 20% of patients [2], which aligns with the absence of systemic symptoms in this case. We report a rare case of primary splenic FL to illustrate the diagnostic challenges and management of this unusual presentation.

## Case presentation

A woman in her late 60s with no significant medical history presented with progressive abdominal distension, early satiety, reduced appetite, fatigue, and unintentional weight loss over three months. She denied fever, night sweats, peripheral edema, urinary symptoms, or dyspnea. Family history was notable for lung carcinoma in her father and liver carcinoma in her mother. On examination, she was afebrile and hemodynamically stable. The patient had marked splenomegaly, with the spleen palpable approximately 20 cm below the left costal margin. No hepatomegaly, ascites, or peripheral lymphadenopathy were present. Cardiopulmonary examination was unremarkable.

Baseline laboratory investigations (Table 1) showed mild thrombocytopenia but were otherwise within normal limits. Hemoglobin was 12 g/dL, WBC count 4,770/mm³, and platelet count 127,000/mm³. Peripheral blood smear showed normocytic, normochromic red cells with no abnormal or immature forms. Lactate dehydrogenase (LDH) and uric acid levels were normal. Liver and renal function tests were also normal. An abdominal ultrasound confirmed massive splenomegaly (long axis ~25 cm) with a normal liver and no ascites. Given the isolated splenomegaly with cytopenia, a working diagnosis of a chronic lymphoproliferative disorder with primary splenic involvement was considered.

**Table 1:** Baseline laboratory investigations. Mild thrombocytopenia was noted, while all other parameters were within normal limits. LDH: Lactate Dehydrogenase; AST: Aspartate Aminotransferase; ALT: Alanine Aminotransferase.

Parameter	Result	Reference Range
Hemoglobin	12.0 g/dL	12-16 g/dL
Total leukocyte count	4,770/mm³	4,000-11,000/mm³
Platelet count	127,000/mm³	150,000-400,000/mm³
Peripheral smear	Normocytic, normochromic RBCs; no atypical cells	-
Serum LDH	180 U/L	125-220 U/L
Uric acid	4.8 mg/dL	2.4-6.0 mg/dL
AST	22 U/L	0-35 U/L
ALT	18 U/L	0-35 U/L
Total bilirubin	0.8 mg/dL	0.2-1.2 mg/dL
Creatinine	0.9 mg/dL	0.6-1.2 mg/dL

Fluorodeoxyglucose positron emission tomography/computed tomography (FDG PET/CT) demonstrated an enlarged spleen measuring approximately 25 cm in length with diffusely increased metabolic activity (SUV ~6). There were no FDG-avid lymph nodes in the neck, axillae, mediastinum, abdomen, or pelvis, and no other organ involvement was seen. Bone marrow biopsy from the iliac crest revealed normocellular trilineage marrow with a single small lymphoid aggregate. Immunohistochemistry on the marrow sample showed the aggregate was positive for BCL2 but negative for CD10 and BCL6. CD20 highlighted a small cluster of B lymphocytes, while CD3 highlighted interspersed T lymphocytes, consistent with a benign reactive lymphoid aggregate rather than definitive marrow involvement by lymphoma. Given the inconclusive bone marrow findings and persistent concern for isolated splenic lymphoma, a therapeutic and diagnostic splenectomy was performed.

**Figure 1:**
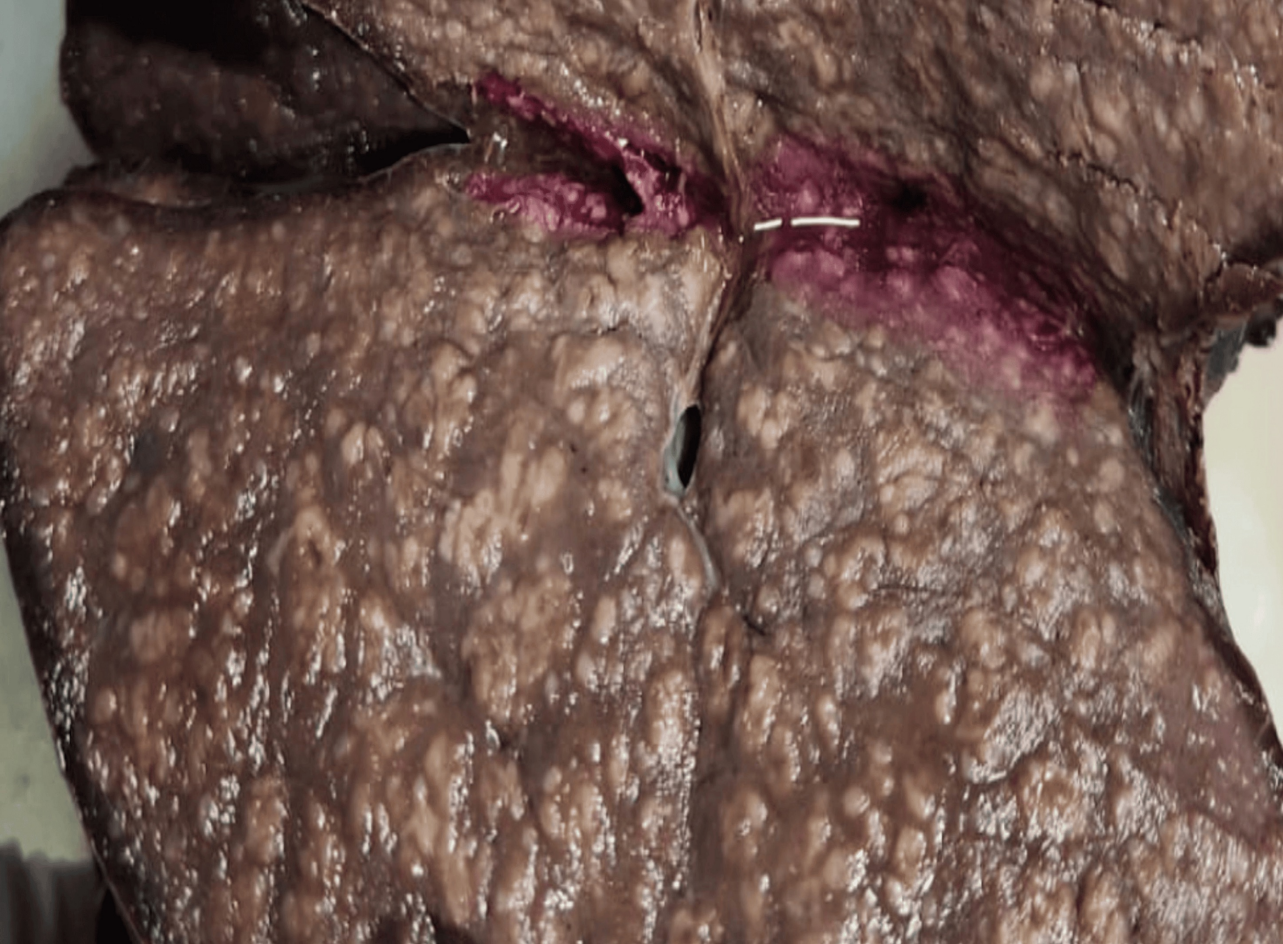
Gross image of the cut surface of the spleen. The resected spleen shows diffuse nodularity, representing innumerable tumor nodules replacing the splenic parenchyma.

At surgery, the spleen was massively enlarged, measuring 33 × 16 × 6 cm and weighing 1,200 g. On gross section, the splenic parenchyma was studded with numerous confluent pale nodules (Figure 1).

**Figure 2:**
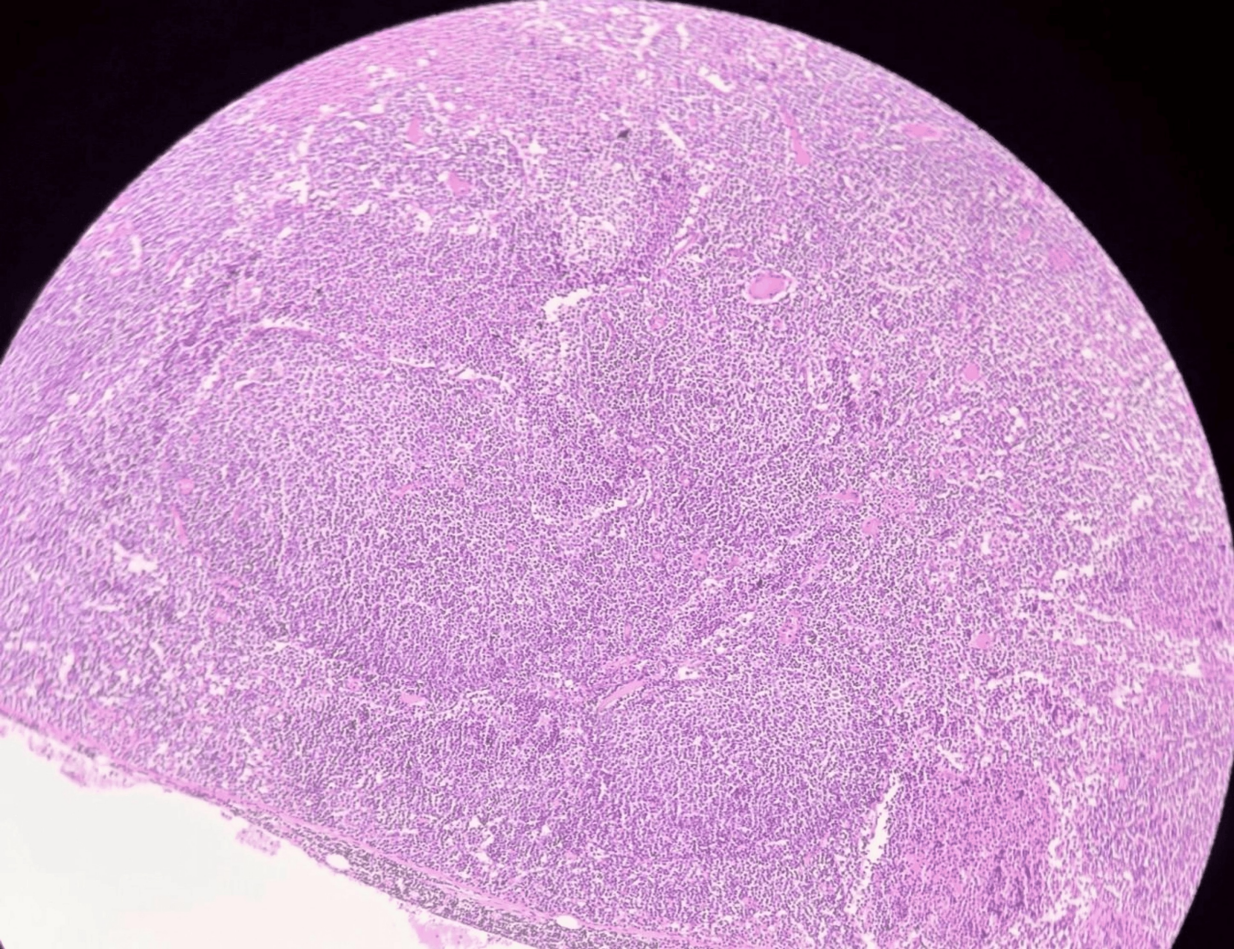
Histopathology of the splenic lesion (H&E stain, 100×, full-field view). The spleen exhibits closely packed abnormal lymphoid follicles lacking mantle zones and germinal center polarization into dark and light zones, features characteristic of follicular lymphoma. Occasional residual splenic red pulp is present between follicles.

Histopathological examination of the spleen (Figure 2) showed a follicular-patterned lymphoid neoplasm: the white pulp was replaced by closely packed neoplastic follicles lacking discernible mantle zones or germinal center polarization. The neoplastic follicles were composed of a mixture of small cleaved lymphocytes (centrocytes) and a few larger centroblasts, consistent with low-grade FL. The splenic red pulp was largely free of tumor involvement. No evidence of diffuse large B-cell lymphoma or marginal zone morphology was seen.

**Figure 3:**
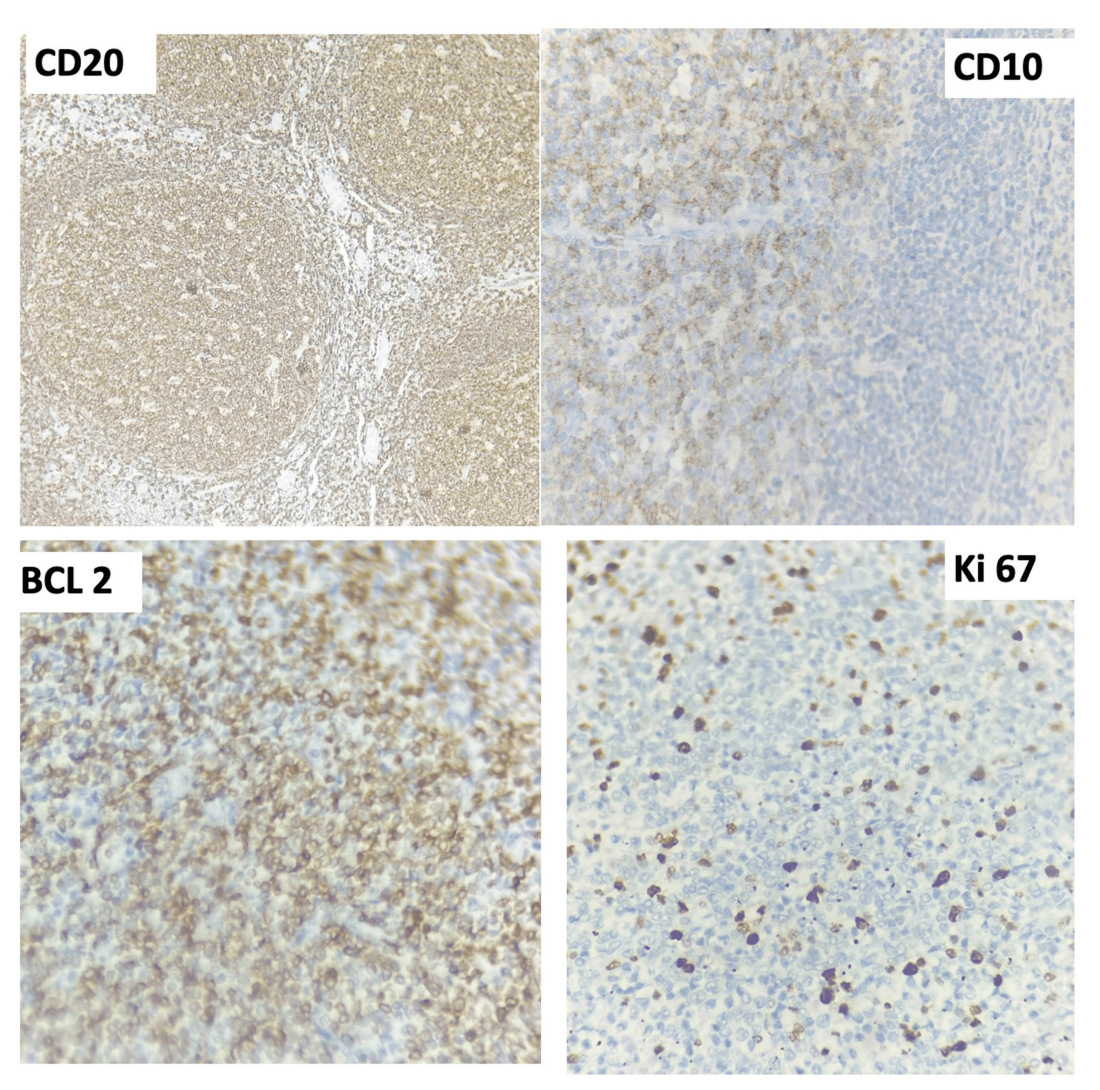
Immunohistochemical findings from the splenic follicular lymphoma. (A) CD20 staining highlights a diffuse population of B cells filling the neoplastic follicles. (B) BCL2 immunostain is strongly positive in the tumor follicles, confirming inappropriate BCL2 expression in neoplastic germinal centers. (C) Ki-67 proliferation index is approximately 20% within the neoplastic follicles, consistent with a low-grade (indolent) lymphoma. All images at 400× magnification. CD10 and BCL6 were also positive in tumor cells, while c-MYC and MUM1 were negative.

Immunohistochemistry confirmed the follicular center cell immunophenotype (Figure 3). The neoplastic B cells were strongly positive for CD20 and CD10, with diffuse expression of BCL2 and BCL6 in the neoplastic follicles. Stains for markers of activation and differentiation were negative in the tumor cells (c-MYC negative; MUM1/IRF4 negative). The Ki-67 proliferation index was approximately 20%, appropriate for indolent (grade 1-2) FL. No evidence of a high-grade transformed component was observed.

Prognostication in follicular lymphoma is commonly performed using the Follicular Lymphoma International Prognostic Index (FLIPI), which incorporates age, stage, hemoglobin, LDH, and number of nodal areas. In this patient, disease was Ann Arbor stage I (spleen only), LDH and hemoglobin were normal, and age was >60 years. Thus, the calculated FLIPI score was 1, corresponding to a low-risk category. Given the unique primary splenic presentation and complete cytoreduction by splenectomy, the prognostic significance of FLIPI in this setting may be limited, but it nonetheless suggested a favorable outcome. She was started on therapy with the anti-CD20 monoclonal antibody rituximab (375 mg/m² intravenously) once weekly for four doses, followed by maintenance rituximab every two months. At the six-month follow-up, she was asymptomatic, with no clinical or radiographic evidence of disease recurrence, and her blood counts had normalized.

## Discussion

Primary splenic lymphoma (PSL) is an uncommon malignancy, and its definition remains debated. The strictest definition confines PSL to disease limited to the spleen and splenic hilar lymph nodes, with no recurrence after splenectomy. Broader criteria also allow concurrent bone marrow involvement in the absence of widespread nodal disease. Using the strict definition, PSL accounts for ~1% of non-Hodgkin lymphomas. Massive splenomegaly has a broad differential diagnosis, including congestive causes (e.g., portal hypertension), reactive causes (e.g., infections such as malaria, granulomatous diseases such as sarcoidosis), and hematologic malignancies. In the context of PSLs, splenic marginal zone lymphoma (SMZL) is the most common subtype, followed by diffuse large B-cell lymphoma and others, whereas FL comprises only a small minority (≈5%) [3]. The rarity of primary splenic FL contributes to diagnostic difficulty. Our patient’s presentation, with isolated splenomegaly, minimal symptoms, and no lymphadenopathy, is characteristic of the indolent nature of this disease [4].

Histologically, FL in the spleen may have distinctive features. Mollejo M et al. described two clinicopathologic variants of splenic FL [5], which are summarized in Table 2. The first resembles conventional nodal FL, characterized by the classic t(14;18) IGH/BCL2 translocation and strong expression of germinal center markers (CD10, BCL6, BCL2). The second variant is a higher-grade form with an increased proliferative index, often lacking the t(14;18) translocation and BCL2 expression; this form tends to be truly confined to the spleen. Our case aligns with the classic variant.

**Table 2:** Variants of primary splenic follicular lymphoma. In FL, a Ki-67 index of less than 40% is considered low grade, whereas more than 40% is considered high grade. Our patient’s index (~20%) placed her in the indolent, low-grade category. IHC: Immunohistochemistry; FL: Follicular Lymphoma; IGH: Immunoglobulin Heavy Chain; BCL2: B-cell Lymphoma 2; CD10: Cluster of Differentiation 10; BCL6: B-cell Lymphoma 6.

Variant	Morphology & IHC Profile	Genetic Features	Ki-67 Index	Clinical Features
Classic / nodal-like	Follicular pattern; CD10+, BCL6+, BCL2+	t(14;18) IGH/BCL2 translocation common	Usually low (<40%)	Indolent course; resembles nodal FL
High-grade splenic variant	Often diffuse areas; may lack BCL2; variable CD10/BCL6	Frequently lacks t(14;18)	High (>40%)	More aggressive; confined to spleen; higher relapse risk

Another diagnostic challenge is evaluating bone marrow involvement. Benign (reactive) lymphoid aggregates in bone marrow are typically small, well-circumscribed, non-paratrabecular, and lack cytological atypia, whereas malignant aggregates in FL are often paratrabecular [6]. Immunohistochemistry can aid distinction: lymphoma cells in FL usually co-express BCL2, CD10, and BCL6, but BCL2 alone is not specific, as reactive aggregates may also show positivity [7]. This underscores why splenectomy was critical in establishing a definitive diagnosis in this case.

Optimal management of primary splenic FL follows general FL principles. In a national series, splenectomy was the initial therapy in 77% of splenic FL patients, and nearly half required no immediate systemic therapy. However, relapse can occur, and adjuvant treatment improves remission duration. Rituximab, an anti-CD20 monoclonal antibody, is a mainstay of FL management. Maintenance rituximab prolongs progression-free survival, though it is not curative [8]. Our patient’s favorable outcome with rituximab monotherapy is consistent with published literature.

## Conclusions

PSFL is an uncommon cause of isolated splenomegaly, but it is an important diagnostic consideration in patients with unexplained massive splenic enlargement. This case illustrates several key points. First, in the absence of lymphadenopathy or clear evidence of disseminated disease, diagnosing the cause of splenomegaly can be challenging; bone marrow findings may be nondiagnostic, and splenectomy can be indispensable in establishing a definitive diagnosis. Second, careful pathological analysis (including appropriate immunohistochemical panels) is required to distinguish primary splenic FL from other splenic lymphoma subtypes and from benign reactive conditions. Third, splenectomy not only provides tissue for diagnosis but also offers immediate therapeutic benefit by debulking the disease and ameliorating symptoms of hypersplenism. Finally, the addition of systemic therapy with rituximab in this setting can deepen remission and delay progression, thereby improving the patient’s quality of life. Early recognition of this rare presentation and a multidisciplinary approach, incorporating gastroenterologists, hematologists, pathologists, radiologists, and surgeons, are essential to achieve optimal outcomes. While the prognosis for limited-stage FL is generally favorable, long-term surveillance is necessary given the risk of late relapses or transformation.
